# Detection of *Mycoplasma mycoides* subsp. *mycoides* SC in bronchoalveolar lavage fluids of cows based on a TaqMan real-time PCR discriminating wild type strains from an *lppQ*^−^ mutant vaccine strain used for DIVA-strategies

**DOI:** 10.1016/j.mimet.2010.03.025

**Published:** 2010-06

**Authors:** Edy M. Vilei, Joachim Frey

**Affiliations:** Research Unit, Institute of Veterinary Bacteriology, Vetsuisse Faculty, University of Bern, Länggass-Strasse 122, P.O. Box, 3001 Bern, Switzerland

**Keywords:** *Mycoplasma mycoides* subsp. *mycoides* SC, TaqMan real-time PCR, Bronchoalveolar lavage fluids, *lppQ*^−^ mutant vaccine strain, DIVA

## Abstract

Contagious bovine pleuropneumonia (CBPP) is the most serious cattle disease in Africa, caused by *Mycoplasma mycoides* subsp. *mycoides* small-colony type (SC). CBPP control strategies currently rely on vaccination with a vaccine based on live attenuated strains of the organism. Recently, an *lppQ*^−^ mutant of the existing vaccine strain T1/44 has been developed (Janis et al., 2008). This T1*lppQ*^−^ mutant strain is devoid of lipoprotein LppQ, a potential virulence attribute of *M. mycoides* subsp. *mycoides* SC. It is designated as a potential live DIVA (Differentiating Infected from Vaccinated Animals) vaccine strain allowing both serological and etiological differentiation. The present paper reports on the validation of a control strategy for CBPP in cattle, whereby a TaqMan real-time PCR based on the *lppQ* gene has been developed for the direct detection of *M. mycoides* subsp. *mycoides* SC in ex vivo bronchoalveolar lavage fluids of cows and for the discrimination of wild type strains from the *lppQ*^−^ mutant vaccine strain.

## 1 Introduction

Contagious bovine pleuropneumonia (CBPP) caused by *Mycoplasma mycoides* subsp. *mycoides* small-colony type (SC), a bacterium belonging to the class Mollicutes, is a major constraint to cattle production in areas of eastern, central and western Africa, either as an endemic or re-emerging disease or in epidemic form (Windsor, 2000; Miltiadou et al., 2009). CBPP is currently considered one of the main stumbling blocks to the growth of the livestock industry on the African continent. Yearly losses directly or indirectly due to CBPP in Africa are estimated to be around 2 billion US dollars (Food and Agriculture Organization of the United Nations, 2004). Control of CBPP is therefore important as a way to salvage the losses and to ensure cattle owners of an income (Tambi et al., 2006). Current strategies to control CBPP rely on local quarantine of affected herds, antibiotic treatment of clinically diseased animals, or vaccination with a vaccine based on live attenuated strains.

Efficient control of CBPP not only requires more efficient vaccine strains (Thiaucourt et al., 2000, 2003; Thiaucourt, 2002; Wesonga et al., 2003; Mbulu et al., 2004; Pilo et al., 2007; Bischof et al., 2009), but also reliable diagnostic strategies that allow differentiating infected from vaccinated animals (DIVA). Lipoprotein LppQ is a particular membrane located protein of *M. mycoides* subsp. *mycoides* SC as it is serologically specific to this organism. It has a particularly strong antigenic N-terminal part that is located on the outer surface of the membrane (Bonvin-Klotz et al., 2008) and that elicits a specific and early immune response in naturally and experimentally infected animals (Bruderer et al., 2002). The C-terminal part of LppQ possesses an integral membrane structure built up of repeated units that are suspected to be involved in membrane anchoring (Abdo et al., 2000; Bonvin-Klotz et al., 2008). A recent study has shown that cattle immunized with the recombinant peptide comprising the extracellular N-terminal part of LppQ, using different adjuvant methods, were significantly more susceptible to infection with a virulent *M. mycoides* subsp. *mycoides* SC strain than cattle that were not vaccinated with LppQ (Nicholas et al., 2004). Hence, LppQ — which is devoid of protective T-cell epitopes (Dedieu et al., 2009) — appeared to exacerbate the effects of CBPP. Thus, an attenuated mutant lacking *lppQ* is likely to be a valuable second-generation vaccine and is expected to give lower side effects.

In the view to develop new efficacious and safe vaccine strains, the gene *lppQ* was inactivated by genetic engineering in the currently used live vaccine strain T1/44 using a transposon-based approach (Janis et al., 2008). In the present work, we aimed to develop a diagnostic tool for detection of *M. mycoides* subsp. *mycoides* SC in the lungs of CBPP-affected cattle and which allows discrimination of wild type strains from the recombinant DIVA T1*lppQ*^−^ mutant vaccine strain.

## 2 Materials and methods

### 2.1 Mycoplasma strains, growth conditions and DNA extraction

Strains of mycoplasmas (*n* = 86) used in this study are listed in Table 1. Cells were grown in a standard mycoplasma medium (Axcell Biotechnologies, St. Genis l'Argentière, France) at 37 °C to a density of 10^8^–10^9^ cells/ml or on solid mycoplasma agar medium (Axcell Biotechnologies). Growth and handling of live *M. mycoides* subsp. *mycoides* SC were performed in a biological safety laboratory fulfilling the BL3 containment safety standards. Lysis of mycoplasmas with GES buffer (5 M guanidium thiocyanate, 100 mM EDTA, 0.5% *N*-lauroylsarcosine) and extraction of genomic DNA were performed as previously described (Vilei and Frey, 2001; Pilo et al., 2005; Bischof et al., 2008).

Disruption of *lppQ* in the vaccine T1/44 was achieved by transposon-based genetic engineering to obtain the mutants T1*lppQ*^−^MT1 and T1*lppQ*^−^RSP1 (Janis et al., 2008) (Table 1).

### 2.2 PCR and sequencing strategies

Polymerase chain reaction (PCR) was performed with a DNA thermal cycler Gene Amp PCR System 9600 (Applied Biosystems, Foster City, CA, USA) in a 30-μl reaction mixture [1× reaction buffer B (supplied with FIREPol® DNA polymerase), 2.5 mM MgCl_2_, 250 μM of each dNTP] that contained approximately 50 ng of genomic template DNA, 2.5 U of FIREPol® DNA polymerase (Solis BioDyne, Tartu, Estonia), and 400 nM of oligonucleotide primers MMMLP481 and MMMLP484 (Abdo et al., 2000) for *lppQ* amplification. The mixtures were subjected to 3-min denaturation at 94 °C followed by 35 cycles of amplification with the parameters: 30 s at 94 °C, 30 s at 48 °C and 3 min extension at 72 °C, and a final extension step at 72 °C for 7 min. Amplicons were purified with the High pure PCR product purification kit (Roche Diagnostics, Rotkreuz, Switzerland).

DNA sequence analysis of the purified amplicons was performed with a DNA Sequenator AB 3100 genetic analyzer and the *Taq* dye deoxy terminator cycle sequencing kit (Applied Biosystems) using primers MMMLP481 and MMMLP484 for *lppQ* (Table 2). The assembly of DNA sequences and alignments of sequenced segments were done using the program Sequencher 4.6 (GeneCodes, Ann Arbor, MI, USA). Comparisons of DNA sequences and their deduced amino acid sequences with the EMBL/GenBank database were performed using the BLAST programs blastn, blastx and blastp (Altschul et al., 1990).

### 2.3 Selection of primers and TaqMan probes

**Fig. 1:**
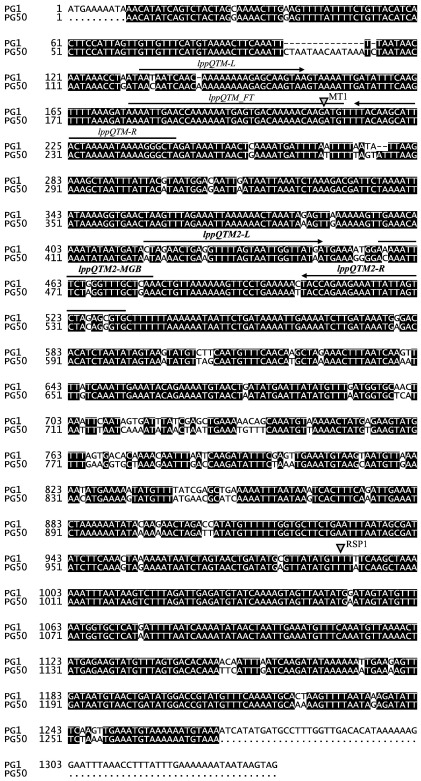
Location of the TaqMan primers and probes within the *lppQ* gene. The sequence of the *lppQ* gene from type strain PG1 (where it is present in two copies) of *M. mycoides* subsp. *mycoides* SC was aligned with the *lppQ* pseudogene sequence from the reference strain PG50 of *M. leachii* using the program DIALIGN 2.2.1 (http://bibiserv.techfak.uni-bielefeld.de/dialign/submission.html), and the alignment was finally elaborated with Boxshade 3.21 (http://www.ch.embnet.org/software/BOX_form.html). Identical nucleotides are shown on black background. The grey arrowheads indicate the site of disruption of *lppQ* in the mutants T1*lppQ*^−^MT1, which produces only 69 N-terminal amino acids of LppQ, and T1*lppQ*^−^RSP1, which still produces about 3/4 of the LppQ polypeptide. The mutant strains were achieved by genetic engineering using a transposon-based approach for the random mutagenesis of vaccine strain T1/44 (Janis et al., 2008). TaqMan primers are indicated by arrows, probes by lines.

Primer Express 2.0 software (Applied Biosystems) was used to design oligonucleotide primers and fluorogenic probe, to be used in a TaqMan platform, specific to the locus of the gene *lppQ* of wild type strains of *M. mycoides* subsp. *mycoides* SC. The software displayed the two primers lppQTM-L and lppQTM-R, and the probe lppQTM_FT with FAM reporter dye and TAMRA quencher affixed on the 5′ and 3′ ends, respectively (Table 2) (Bischof et al., 2006). This TaqMan FT assay can discriminate wild type strains of *M. mycoides* subsp. *mycoides* SC from the recombinant strain T1*lppQ*^−^MT1, as the FT probe is concerned by the integration of the transposon after nucleotide position 208 of the *lppQ* gene (Fig. 1). As strains of *Mycoplasma leachii* — formerly known as *Mycoplasma* sp. bovine group 7 (Manso-Silván et al., 2009) — harbour a DNA fragment that is similar to *lppQ* (Fig. 1), real-time PCR using these oligonucleotides also detected strains of *M. leachii*. Thus, in the optic to select *lppQ*-specific TaqMan primers and probe that can discriminate *M. mycoides* subsp. *mycoides* SC from *M. leachii* (as well as from all other mycoplasmas), a new search was performed by using the TaqMan MGB (minor groove binder) primer and probe design option of Primer Express 2.0. MGB is a non-fluorescent quencher and substitutes TAMRA at the 3′ end of the probe. The sequence of the employed 22-nt probe lppQTM2-MGB is indicated in Table 2. As already reported (Vilei et al., 2007), primers lppQTM2-L and lppQTM2-R (Table 2) specifically amplify a 117-bp fragment from the locus of the gene *lppQ* of *M. mycoides* subsp. *mycoides* SC. All three oligonucleotides of the TaqMan MGB assay, however, match with the disrupted *lppQ* of the DIVA recombinant strain T1*lppQ*^−^MT1 (Fig. 1) and hence cannot discriminate the latter.

### 2.4 Real-time PCR assays

Reactions were performed by using 2.5 µl of samples from each strain or 2.5 µl of 10 min-boiled bronchoalveolar lavage fluids, a 900 nM concentration of each TaqMan primer (Table 2) and 300 nM TaqMan probe (Table 2), and TaqMan Universal PCR Master Mix No AmpErase UNG (Applied Biosystems) in a 25-μl volume. An exogenous Internal Positive Control (IPC) reagent, consisting of 0.5× TaqMan IPC DNA, and 0.5× TaqMan IPC primers and probe (with VIC reporter and TAMRA quencher) (Applied Biosystems), was spiked into each reaction well of the TaqMan MGB assay in order to distinguish true target negatives from PCR inhibition. The concentration of the IPC primers in the PCR reaction was set reasonably lower than that of target primers, so that the amplification efficiency of the target reaction was not compromised. Real-time PCR reactions were run on an ABI 7500 instrument (Applied Biosystems) using the following cycling parameters: after one step at 50 °C for 2 min and at 95 °C for 10 min, 40 cycles of denaturation at 95 °C for 15 s and extension at 60 °C for 1 min were performed. Real-time fluorescence measurements were taken for each sample by using the Sequence Detector software 1.3.1 (Applied Biosystems) and the PCR cycle number at which the fluorescent *lppQ*-signal crossed the cycle threshold limit for each sample (2.5 µl) was recorded as Ct value. The threshold, i.e., the detected limit where the first PCR cycle has a significant increase in fluorescence signal, was adjusted at 0.1 for the TaqMan FT assay and at 0.2 for the TaqMan MGB assay. In both cases, thresholds were moved higher in the exponential phase of amplification than the default threshold values to avoid background noise. The fluorescence emission baseline was calculated from the first 3 to 15 cycles. Each sample was assayed in duplicate (unless otherwise stated), and the assay was repeated if the standard deviation (SD) between the two replicates was greater than 1 Ct.

### 2.5 Standard curves and specificity of the TaqMan assays

Three-day cultures (10^8^–10^9^ cells/ml, see above) of *M. mycoides* subsp. *mycoides* SC strains C305 and 2022 (Table 1) were centrifuged at 36,000 × *g* for 30 min. The pellets were washed once and then resuspended at a density of approximately 5 × 10^10^ cells/ml with the aid of a 27-gauge needle in sterile 0.85% sodium chloride solution. Small aliquots of these mycoplasma suspensions were used to count the number of colony forming units CFU obtained from 10-fold serial dilutions on solid mycoplasma medium (Axcell Biotechnologies). TaqMan standard curves were produced by analyzing serial dilutions of the two strains in lysis buffer (100 mM Tris–HCl, pH 8.5, 0.05% Tween 20, 0.24 mg/ml proteinase K), which contained 1.5 to 1.5 × 10^7^ CFU per reaction (i.e., 600 to 6 × 10^9^ CFU/ml). Samples were mixed for 1 min and incubated for 60 min at 60 °C followed by incubation for 15 min at 95 °C to obtain the lysates as template for PCR reactions in triplicates. The efficiency of the two described TaqMan reactions was calculated considering the slope of their standard curves and using the formula (10^− 1/slope^).

The specificity of the TaqMan assays was evaluated by testing approximately 10 ng of genomic DNA of each mycoplasma (*n* = 86) listed in Table 1, which covered the five members of the *mycoides* cluster that contains *M. mycoides* subsp. *mycoides* SC, *M. leachii*, *M. mycoides* subsp. *capri* — that now groups also strains of the serovar *M. mycoides* subsp. *mycoides* large-colony type (LC) (Manso-Silván et al., 2009) — *Mycoplasma capricolum* subsp. *capricolum*, and *M. capricolum* subsp. *capripneumoniae*, as well as a range of other species covering phylogenetically related species and representatives of other animal species. DNA was quantified spectrophotometrically and by visual comparison after gel electrophoresis and ethidium bromide staining.

### 2.6 Artificial contamination of bronchoalveolar lavage fluid with mycoplasmal preparations

Samples of bronchoalveolar lavages (Baselski and Wunderink, 1994; Miserez et al., 1997) from cattle were contaminated artificially with four consecutively 10-fold diluted suspensions of *M. mycoides* subsp. *mycoides* SC strains C305, O326 and 2022 (Table 1) to reach 2.8 × 10^6^ to 2.8 × 10^9^ CFU/ml. Quantification using the *M. mycoides* subsp. *mycoides* SC-specific TaqMan MGB assay was performed to confirm these CFU values and to assess the diagnostic efficacy of the lipoprotein gene *lppQ*-based real-time PCR for detection of *M. mycoides* subsp. *mycoides* SC in bronchoalveolar lavage fluids of cows.

### 2.7 Nucleotide sequence accession numbers

The nucleotide sequence of the *lppQ* pseudogene from the reference strain PG50 of *M. leachii* has been deposited under the EMBL/GenBank accession number AM158959 (1276 nucleotides in length).

## 3 Results

### 3.1 Standard curve and detection limit of the TaqMan assays

**Fig. 2:**
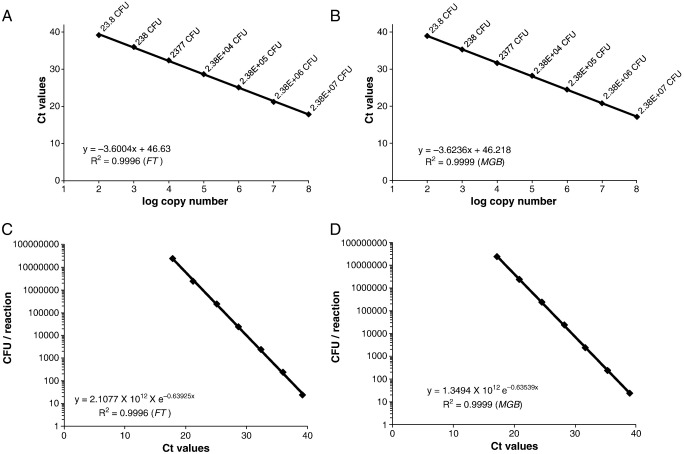
TaqMan PCR amplification of serial 10-fold dilutions of *M. mycoides* subsp. *mycoides* SC in lysis buffer. TaqMan FT (panel A) and MGB (panel B) standard curves generated by the analysis of known amounts of cells (CFU) of strains C305 and 2022. Three data sets for each strain and each dilution were used to generate a common standard curve. The mean Ct values are plotted against the log copy numbers of the standard dilutions. The linear regressions and the coefficients of correlation *R*^2^ were calculated. TaqMan FT (panel C) and MGB (panel D) standard curves for calculation of the amount of *M. mycoides* subsp. *mycoides* SC cells per reaction. The CFU in the reaction tube is plotted against the Ct values obtained with the serial 10-fold dilutions of *M. mycoides* subsp. *mycoides* SC. The obtained equation CFU_reaction_ = 1.3494 × 10^12^ × e^− 0.63539 × Ct^ of the TaqMan MGB assay was used to calculate the load of mycoplasmas in artificially contaminated bronchoalveolar lavage fluids.

The standard curves for *lppQ* amplification (Ct values vs. log_10_(CFU)) generated by 10-fold serial dilutions of the two *M. mycoides* subsp. *mycoides* SC strains C305 and 2022 in lysis buffer were linear over a range of 7 log units, as illustrated by *R*^2^ values of 0.9996 (FT assay) and 0.9999 (MGB assay). The slopes were − 3.6004 (FT assay; Fig. 2A) and − 3.6236 (MGB assay; Fig. 2B), and the efficiencies (10^− 1/slope^) of the TaqMan assays were therefore 1.8956 (FT assay) and 1.8879 (MGB assay), both close to 2 (perfect PCR amplification). The detection limit of both TaqMan assays was at ∼ 15 CFU per reaction, corresponding to ∼ 6000 CFU/ml. The estimated quantities of mycoplasmal cells in clinical samples (see below) were determined using the formulas CFU_reaction_ = 2.1077 × 10^12^ × e^− 0.63925 × Ct^ (FT assay; Fig 2C) and CFU_reaction_ = 1.3494 × 10^12^ × e^− 0.63539 × Ct^ (MGB assay; Fig 2D), generated by linear regressions of the standard curves.

### 3.2 The *lppQ* pseudogene in *M. leachii*

*M. leachii* revealed a pseudogene of *lppQ* (AM158959) with stop codons in all three forward frames, ruling out the possibility of having any lipoprotein production. It has a nucleotide similarity of 84% to the *lppQ* gene of *M. mycoides* subsp. *mycoides* SC, showing totally 18 gaps (Fig. 1). The TaqMan MGB primers and probe (Table 2) were selected from the available *lppQ* sequences from strains of *M. mycoides* subsp. *mycoides* SC so that they do not anneal to *lppQ* of *M. leachii*.

### 3.3 TaqMan specificities

The wild type strains of *M. mycoides* subsp. *mycoides* SC analyzed (*n* = 27) and the mutant vaccine T1*lppQ*^−^RSP1 gave positive signals when 10 ng of genomic DNA were tested in the TaqMan FT assay, yielding a mean Ct value of 19.51 (SD, 0.28). DNA of the mutant vaccine T1*lppQ*^−^MT1 remained negative in this TaqMan FT assay. However, strains of *M. leachii* (*n* = 12) all gave positive signals (Table 1).

In order to differentiate these latter from *M. mycoides* subsp. *mycoides* SC, the TaqMan MGB assay was used. All *M. mycoides* subsp. *mycoides* SC strains analyzed (*n* = 29), including both mutants T1*lppQ*^−^MT1 and T1*lppQ*^−^RSP1 carrying disrupted *lppQ* genes, gave specific signals when 10 ng of genomic DNA were tested in this TaqMan MGB assay, yielding a mean Ct value of 18.92 (SD, 0.22). The wide range of other *Mycoplasma* species (*n* = 57) gave negative reactions by the TaqMan MGB assay (Table 1).

### 3.4 Reproducibility of TaqMan MGB quantification with regard to the CFU counts

Linear curves obtained from the *lppQ*-based TaqMan MGB real-time PCR of bronchoalveolar lavage fluids contaminated artificially with four 10-fold dilutions of *M. mycoides* subsp. *mycoides* SC cultures from strains C305, O326 and 2022 had *R*^2^ values of > 0.999 (not shown). The estimated quantities of mycoplasmal cells in the suspensions were derived from the linear regression of the standard curve of the *M. mycoides* subsp. *mycoides* SC-specific TaqMan MGB assay (see above) and were found to be comparable to the known amount of inoculated cells (CFU), with a maximal divergence of approximately 30% only (Table 3). Using higher concentrations (≥ 2.8 × 10^8^ CFU/ml), the calculated mycoplasmal loads in the suspensions were found to be moderately lower (− 15% to − 30%) compared to the inoculated CFU counts, whereas they were only slightly lower (0% to − 15%) at mycoplasmal concentrations ≤ 2.8 × 10^7^ CFU/ml.

## 4 Discussion

The high specificity and strong antigenicity of LppQ have been already exploited for the development of a robust indirect ELISA test for serological diagnosis and for epidemiological investigations of CBPP (Bruderer et al., 2002). This ELISA test is able to differentiate infected cattle from cattle immunized with the *M. mycoides* subsp. *mycoides* SC DIVA strain T1*lppQ*^−^MT1, as the latter produces no LppQ (Janis et al., 2008). Also several DNA-based assays, which are less burdensome than diagnostic methods based on immunological tests, have already been developed for the efficient detection of *M. mycoides* subsp. *mycoides* SC: these include Southern blotting (Frey et al., 1995; Vilei et al., 1999; Pilo et al., 2003), conventional PCR tests (Bashiruddin et al., 1994; Dedieu et al., 1994; Miserez et al., 1997; Bashiruddin et al., 1999; Persson et al., 1999; Vilei et al., 2000; Vilei and Frey, 2004; Miles et al., 2006) and real-time PCR assays (Gorton et al., 2005; Bischof et al., 2006, 2008; Vilei et al., 2007; Fitzmaurice et al., 2008; Lorenzon et al., 2008).

The current study has evidenced that the real-time PCR assay using primers lppQTM-L and lppQTM-R, and the probe lppQTM_FT (Table 2) in the *lppQ*-based TaqMan FT assay (Bischof et al., 2006) works in such a discriminatory way that the disrupted *lppQ* gene in the LppQ^−^ mutant vaccine T1*lppQ*^−^MT1 is not detected. However, this TaqMan FT assay also recognizes strains of *M. leachii* because the latter harbour an *lppQ* pseudogene that is not expressed due to stop codons in all three forward frames. On the other hand, the second *lppQ*-based real-time PCR assay employed in this study, which uses primers lppQTM2-L and lppQTM2-R, and the probe lppQTM2-MGB (Table 2) in the TaqMan MGB assay (Vilei et al., 2007) may be applied to specifically monitor *M. mycoides* subsp. *mycoides* SC in the lungs of cattle presenting CBPP symptoms. In fact, this TaqMan MGB assay was able to detect all DNAs extracted from *M. mycoides* subsp. *mycoides* SC strains while the wide range of related *Mycoplasma* species, including *M. leachii*, gave no reactions. Another important feature is the sensitivity of both *lppQ*-based TaqMan assays, whereby as less as 6000 mycoplasmal cells/ml can be detected efficiently. While validating the reproducibility of the *M. mycoides* subsp. *mycoides* SC-specific TaqMan MGB assay, the estimated quantities of mycoplasmal cells in the artificially contaminated clinical samples were comparable to the inoculated CFU counts, showing divergences lower than 30%. Sensitivity and specificity of the test under field conditions (living animals) remain to be explored.

In conclusion, the two lipoprotein gene *lppQ*-based TaqMan real-time PCR assays described in this paper constitute a rapid, specific and sensitive test for the detection of *M. mycoides* subsp. *mycoides* SC. The MGB assay is specifically designed to monitor efficiently the mycoplasma in the bronchoalveolar lavage fluids of cattle presenting CBPP symptoms, while the FT assay will be applicable for future differentiation of cattle infected with wild type strains of *M. mycoides* subsp. *mycoides* SC from those immunized with the DIVA vaccine, i.e., the T1*lppQ*^−^MT1 derivative of the classical T1/44 vaccine strain.

## Figures and Tables

**Table 1:** TaqMan results for the 86 strains of mycoplasmas tested in this study.

Species	Strain^a^	Origin	Isolated	Host	TaqMan	
					FT^b^	MGB^b^
*M. mycoides* subsp. *mycoides* SC	PG1		1931	Cattle/type strain	+	+
	2059	Spain	1984	Cattle/lung	+	+
	B773/125	Portugal	1991	Cattle/semen	+	+
	C305	Portugal	1993	Goat/lung	+	+
	O326	Portugal	1993	Sheep/milk	+	+
	PO 1967	France	1967	Cattle/lung	+	+
	PO 2	France	1980	Cattle/lung	+	+
	2022	France	1984	Cattle/lung	+	+
	L2	Italy	1993	Cattle/lung	+	+
	402	Italy	1990	Cattle/lung	+	+
	6479	Italy	1992	Cattle/lung	+	+
	Afadé	Cameroon	1968	Cattle/lung	+	+
	87137-9	Burkina Faso	1987	Cattle	+	+
	Filfili	Senegal	Pre-1988	Cattle	+	+
	Fatick	Senegal	1968	Cattle	+	+
	B17	Chad	1967	Zebu	+	+
	9050-529/1	Ivory Coast	1990	Cattle	+	+
	91130	Central African Republic	1991	Cattle	+	+
	94111	Rwanda	1994	Cattle	+	+
	95014	Tanzania	1995	Cattle	+	+
	T1/44	Tanzania	1952	Cattle/vaccine strain	+	+
	T1*lppQ*^−^MT1	DIVA mutant	2008	Transposon-based random mutagenesis	−	+
	T1*lppQ*^−^RSP1	Recombinant mutant	2008	Transposon-based random mutagenesis	+	+
	T1/Sr50	Tanzania	1952	Cattle/vaccine strain	+	+
	KH_3_J	Sudan	1940	Cattle/vaccine strain	+	+
	Gladysdale	Australia	Pre-1964	Cattle	+	+
	DVZ	Australia	1965	Cattle	+	+
	R575	Australia	1965–1968	Cattle	+	+
	V5	Australia	1936	Cattle/vaccine strain	+	+
*Mycoplasma leachii*	PG50	Australia	1963	Cattle/joint/reference strain	+	−
	B5415	Portugal		Cattle	+	−
	CP291	Portugal		Goat/lung	+	−
	PAD3186	India	1993	Goat/milk	+	−
	FRD424	India	1993	Goat/milk	+	−
	Calf 1	Nigeria		Cattle/blood	+	−
	D318b	Germany		Cattle/semen	+	−
	C2306	Portugal		Cattle	+	−
	D424	Germany		Cattle/preputium	+	−
	QR1/92	Australia			+	−
	4055	France		Cattle/lung	+	−
	B144P	USA	1956	Cattle/joint	+	−
*M. mycoides* subsp. *capri*	PG3	Turkey	1950	Goat/type strain	−	−
	N108	Nigeria	1977	Goat	−	−
	Capri L	France	1975	Goat	−	−
	9139/11-91	Turkey	1991		−	−
	WK354/80	Switzerland	1980	Goat	−	−
	213	India	1984	Goat	−	−
	Y-goat	Australia	1956	Goat/type strain (serovar *Mmm*LC)	−	−
	152/93	Grand Canary	1993	Goat	−	−
	LC8065	France		Goat	−	−
	D2482/91	Switzerland	1991	Goat	−	−
	950010	France	1995	Goat	−	−
	D2083/91	Switzerland	1991	Goat	−	−
	CP271	Portugal	1991	Goat	−	−
	D2503	Switzerland		Goat	−	−
*M. capricolum* subsp. *capricolum*	California kid	USA	1955	Goat/type strain	−	−
	173/87	Greece	1987	Sheep	−	−
	6443.90	France	1990	Goat	−	−
*M. capricolum* subsp. *capripneumoniae*	F38	Kenya	1976	Goat/type strain	−	−
	9081-487p	Oman	1990	Goat	−	−
	Gabès	Tunisia	1981	Goat	−	−
*M. bovis*	PG45	USA	1962	Cattle/milk/type strain	−	−
	ML1	France	Pre-1999	Rabbit/lung	−	−
	120/81	Germany	1980–1990	Cattle/milk	−	−
	221/89	Germany	1980–1990	Cattle/milk	−	−
	86p	Belgium	1990–2000	Cattle/milk	−	−
	39G	Belgium	1990–2000	Cattle/bronchoalveolar lavage fluid	−	−
	0435	Belgium	1990–2000	Cattle/bronchoalveolar lavage fluid	−	−
	9585	Belgium	1990–2000	Cattle/bronchoalveolar lavage fluid	−	−
	2610	UK	1990–2000	Cattle/joint fluid	−	−
	2138	UK	1990–2000	Cattle/milk	−	−
	2960	UK	1990–2000	Cattle/lung	−	−
*M. bovirhinis*	PG43		1967	Cattle/respiratory tract/type strain	−	−
	O/D1467	Switzerland	2007	Cattle/bronchus	−	−
	IMD1656	Switzerland	2008	Cattle/bronchus	−	−
*M. bovigenitalium*	PG11	United Kingdom		Cattle/type strain	−	−
	2D	Australia	1974	Sheep (former serogroup 11 strain)	−	−
*M. bovoculi*	RF20391			Cattle	−	−
*M. agalactiae*	PG2	Spain	1973	Goat/type strain	−	−
	3990	France			−	−
	5725	France	1990	Sheep	−	−
*M. putrefaciens*	KS1	USA		Goat/type strain	−	−
*M. ovipneumoniae*	Y98			Sheep/respiratory tract/type strain	−	−
*M. conjunctivae*	HRC/581	USA	1972	Sheep/type strain	−	−
*M. hyopneumoniae*	J		1965	Swine/type strain	−	−
*M. arginini*	G230			Mouse/brain/type strain	−	−

a Collections: the strains were obtained from the Australian Animal Health Laboratory (AAHL), Geelong, Victoria, Australia; Agence Française de Sécurité Sanitaire des Aliments (AFSSA), Lyon, France; BGVV, Jena, Germany; CIRAD-EMVT, Montpellier, France; Faculdad de Veterinaria, Universidad de Las Palmas, Spain; INRA, Villenave d'Ornon, France; Institute of Veterinary Bacteriology, University of Bern, Switzerland; Laboratório Nacional de Investigação Veterinária, Lisboa, Portugal; Laboratoire de Pathologie Bovine, Lyon, France; National Collection of Type Cultures (NCTC), PHLS, London, UK; National Veterinary Institute, Uppsala, Sweden; and Faculty of Veterinary Medicine, University of Liège, Belgium.

b As determined in this study by real-time PCR of approximately 10 ng of genomic DNA. FT, TaqMan assay with the *lppQ*-based FAM probe (Bischof et al., 2006); MGB, TaqMan assay with the *lppQ*-based minor groove binding probe (Vilei et al., 2007).

**Table 2:** Primers and probes used for TaqMan PCR assays deduced from the *lppQ* gene sequence of *M. mycoides* subsp. *mycoides* SC type strain PG1.

Primer	Sequence (5′–3′)	Position^a^
*lppQ*-based TaqMan FT PCR (Bischof et al., 2006)		
lppQTM-L	AATAATCAACAAAAAAAAGAGCAAGTAAGTAA	1166019–1166050; 1189776–1189807
lppQTM-R	TAGCCCTTTTATTTTTAGTAATGCTTGTAA	1166144–1166115; 1189901–1189872
lppQTM_FT	ACATCTTGTTTTTGTCACTCATTTTTTGGTTCAATTTT	1166113–1166076; 1189870–1189833
*lppQ*-based TaqMan MGB PCR (Vilei et al., 2007)		
lppQTM2-L	CTAGAACTGAGGTTTTAGTAATTGGTTATGA	1166317–1166347; 1190074–1190104
lppQTM2-R	CACGCTCTAGACTAATAATTTCTTCTGGTA	1166433–1166404; 1190190–1190161
lppQTM2-MGB	AAAAATTTCTGGGTTTGCTCAA	1166357–1166378; 1190114–1190135

a Based on nucleotide sequence NC_005364, the complete genome of *M. mycoides* subsp. *mycoides* SC type strain PG1 (Westberg et al., 2004). The two copies of *lppQ* in PG1 span nt 1165902–1167239 and nt 1189659–1190996. Note that *lppQ* occurs only in one copy in all other *M. mycoides* subsp. *mycoides* SC strains (Bischof et al., 2006).

**Table 3:** Comparison between TaqMan MGB assay-calculated mycoplasmal loads in artificially contaminated bronchoalveolar lavage fluids and inoculated CFU counts.

Inoculated^a^ (CFU_reaction_)	C305		O326		2022	
	Load_reaction_^b^	*Δ*(%)^c^	Load_reaction_^b^	*Δ*(%)^c^	Load_reaction_^b^	*Δ*(%)^c^
7 × 10^6^	5.52 × 10^6^ (± 3.72 × 10^5^)	− 21.08	4.93 × 10^6^ (± 3.76 × 10^5^)	− 29.61	5.23 × 10^6^ (± 8.94 × 10^5^)	− 25.23
7 × 10^5^	5.94 × 10^5^ (± 3.47 × 10^4^)	− 15.16	5.74 × 10^5^ (± 2.58 × 10^4^)	− 18.07	5.57 × 10^5^ (± 1.03 × 10^5^)	− 20.38
7 × 10^4^	6.19 × 10^4^ (± 5280)	− 11.64	6.41 × 10^4^ (± 5180)	− 8.50	6.51 × 10^4^ (± 3801)	− 7.03
7 × 10^3^	6524 (± 1260)	− 6.80	6974 (± 250.7)	− 0.37	6864 (± 339.2)	− 1.94

a CFU counts present in 2.5 μl of bronchoalveolar lavage fluids contaminated artificially with 10-fold diluted suspensions of the three strains C305, O326 and 2022.

b Mycoplasmal loads obtained by applying the formula load_reaction_ = 1.3494 × 10^12^ × e^− 0.63539 × Ct^ generated by the linear regression of the standard curve from the *M. mycoides* subsp. *mycoides* SC-specific TaqMan MGB assay. Four data sets for each strain and each dilution were used.

c Percent difference between calculated mycoplasma loads and inoculated CFU counts.
